# Phage therapy for intestinal infections: efficacy, challenges, and future directions in translational research

**DOI:** 10.3389/fmicb.2025.1672198

**Published:** 2025-12-03

**Authors:** Xiaoqing Wang, Jingjing Li, Zimo Ge, Junhao Fan, Dongdao Ma, Huiru Cao, Jiawen Shen, Yange Wang, Zhonghua Liu, Salwa E. Gomaa, Xianghui Li, Xinying Ji, Tieshan Teng

**Affiliations:** 1 School of Basic Medical Sciences, Henan University, Kaifeng, China; 2 Faculty of Pharmacy, Department of Microbiology and Immunology, Zagazig University, Zagazig, Egypt; 3 Henan Provincial Research Center of Engineering Technology for Nuclear Protein Medical Detection, Zhengzhou Health College, Zhengzhou, Henan, China; 4 Henan International Joint Laboratory for Nuclear Protein Regulation, Department of Nuclear Medicine, The First Affiliated Hospital, Henan University College of Medicine, Kaifeng, Henan, China

**Keywords:** phage therapy, intestinal infections, antimicrobial resistance, microbiome modulation, translational medicine

## Abstract

Phage therapy has emerged as a promising alternative to conventional antibiotics for combating intestinal bacterial infections, especially in the era of rising antimicrobial resistance. Despite its therapeutic potential, the clinical translation of phage therapy remains hindered by limited large-scale trial data and incomplete mechanistic understanding. This review systematically evaluates the efficacy of phage therapy in animal models of intestinal diseases, encompassing bacterial infection-induced diarrhea (e.g., cholera, typhoid fever), bacterial enteritis, and sepsis. By synthesizing evidence from bacterial colonization assays, histopathological analyses, and disease severity assessments, we highlight features such as phage-mediated pathogen clearance, changes in inflammatory factors, and intestinal pathology. Furthermore, challenges including phage selection difficulties, host specificity issues, and safety considerations are discussed, along with future research directions aimed at bridging the gap between experimental models and clinical applications.

## 1 Introduction

Despite the early discovery of bacteriophages' antimicrobial properties a century ago, phage therapy remained largely marginalized during the antibiotic era, overshadowed by the remarkable efficacy and convenience of conventional antibiotics (D'herelle, 1931). However, the escalating crisis of antibiotic misuse has reignited interest in phage-based interventions. Two major challenges underscore this urgency: (1) the accelerated emergence of multidrug-resistant (MDR) pathogens, which severely limits treatment options, and (2) the collateral damage to commensal microbiota, where antibiotic-induced dysbiosis compromises colonization resistance and exacerbates opportunistic infections (Ranjbar et al., 2022). Recent decades have witnessed transformative progress in phage research, with clinical evidence now robustly supporting its therapeutic potential (Wang et al., 2020; Zheng et al., 2023). In a 2008–2022 retrospective cohort of 100 consecutive refractory infections, personalized bacteriophage therapy achieved clinical improvement in 77.2 % (88/114) of episodes and eradicated the targeted pathogen in 61.3 % (65/106) (Pirnay et al., 2024). These findings not only validate historical observations but also offer a framework for integrating phage biology into modern antimicrobial stewardship.

The human gut microbiota constitutes an extraordinarily complex ecosystem, harboring a diverse array of symbiotic bacteria, viruses, and yet-to-be-identified microorganisms (Allaband et al., 2019). Metagenomic sequencing has revealed over 140,000 intestinal phage genomes and more than 1,000 pathogenic bacterial genomes in the human gut (Sunagawa et al., 2013; Camarillo-Guerrero et al., 2021). Concurrently, extensive research has established a link between gut microbiota dysbiosis and human diseases, with phages emerging as key modulators in these interactions (Khan Mirzaei et al., 2020; Hannigan et al., 2018; Nakatsu et al., 2018). While the exact mechanisms underlying phage-mediated disease modulation remain incompletely elucidated, accumulating evidence from *in vitro* studies, animal models, and clinical observations supports the therapeutic potential of phage therapy (Ott et al., 2017; Merabishvili et al., 2012).

**Figure 1:**
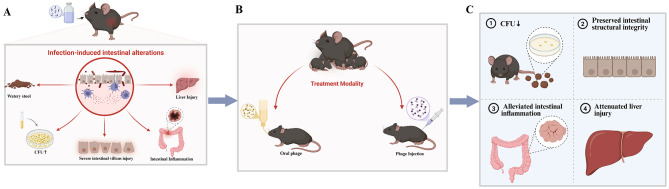
**(A)** Intestinal pathological changes and symptoms induced by pathogenic bacterial infection; **(B)** representative phage treatment regimens in animal models; **(C)** improvement of intestinal pathology and symptoms in mice following phage intervention. Created using Biorender, licensed under Academic License.

Herein, we review common intestinal disease models and their corresponding phage therapy experiments, categorizing and discussing relevant studies to provide a theoretical basis for the application of phage therapy in treating human intestinal diseases (Figure 1).

## 2 Phage-targeted therapy for diarrheal diseases

Diarrhea remains a leading cause of gastrointestinal morbidity worldwide, with bacterial etiologies representing a major public health concern (GBD 2021 Diarrhoeal Diseases Collaborators, 2025). Clinically, bacterial diarrhea manifests as acute-onset, high-frequency watery stools, often accompanied by fluid-electrolyte imbalances and compromised intestinal function (Shankar and Rosenbaum, 2020). Notable bacterial pathogens responsible include Shigella species, enteropathogenic *Escherichia coli, Listeria monocytogenes* and toxigenic *Clostridioides difficile* (Colston et al., 2025; Hensen et al., 2025).

### 2.1 Phage therapy for *Shigella*-associated diarrhea

Diarrheal disease caused by *Shigella spp*. manifests as a severe gastrointestinal infection, characterized by bloody or mucopurulent stools and high mortality in severe cases. Globally, Shigella is estimated to cause approximately 188 million infections and 164,000 deaths annually (Kotloff et al., 2018). In resource-limited settings, it represents the leading etiology of invasive (bloody) diarrhea among children under 5 years of age, with an incidence rate of 0.34 episodes per child-year reported in communities within the Peruvian Amazon (Kosek et al., 2008; Liu et al., 2016). Notably, the 2016 Shigella outbreak in Michigan, which resulted in 177 cases with an 18% hospitalization rate over 8 months across two counties, was the region's most severe in 30 years (Mcclung et al., 2020).

A murine model study investigating *S. sonnei* infection comprehensively validated the prophylactic and therapeutic potential of phage therapy (Mai et al., 2015). In this experiment, mice were orally challenged with 10^8^ colony-forming units (CFU) of *S. sonnei* and administered phages (10^9^ plaque-forming units, PFU) either pre-or post-infection. Notably, all phage treatment regimens drastically suppressed *S. sonnei* colonization in the gastrointestinal tract, including the feces, cecum, and ileum. Within 72 h, phage-treated mice (both pre- and post-infection) demonstrated complete bacterial clearance, whereas persistent colonization was maintained in untreated controls. Strikingly, phage therapy outperformed ampicillin, achieving rapid pathogen clearance within 24–48 h-a critical advantage in acute infections. Over the 28-day monitoring period, phage-treated mice maintained physiological stability, as evidenced by steady body weight, normal leukocyte counts, and absence of diarrheal symptoms. Furthermore, histopathological analysis confirmed no adverse effects in vital organs (heart, brain, liver, kidneys), underscoring the safety profile of this approach (Table 1).

**Table 1:** Case reports of phage therapy for diarrheal diseases.

**Treatment agent**	**Model**	**Outcomes**	**References**
FFT	Human	All patients have returned to normal bowel habits within 6 months	D'herelle, 1931
phiCDHM1-6, phiCDHS1	Hamster	Diarrhea symptoms delayed for 33 h	Kaper et al., 2004
EPEC phage	Mouse	Mice treated with phages alone showed normal weight gain after 10 days	Bruttin and Brüssow, 2005
ListShield	Mouse	The treatment group maintained normal weight	Mai et al., 2010
GJ1, GJ2, GJ3, GJ4, GJ5, GJ6, GJ7	Pig	Percentage of *E. coli* excretion had decreased by 80%	Vahedi et al., 2018
ShigActive™	Mouse	After 72 h, all experimental mice showed no *S. sonnei* and diarrhea symptoms	Mcclung et al., 2020
T4	Human	Fecal phage was detected in approximately 50% of the subjects after 1 day	Gana et al., 2024

### 2.2 Phage therapy for *E. coli*-associated diarrhea

Although *E. coli* typically colonizes the healthy human gut as a commensal microorganism (Kaper et al., 2004), pathogenic strains continue to contribute significantly to diarrheal morbidity and mortality, particularly in resource-limited regions such as Asia, Africa, and Latin America (Chua et al., 2021). The global burden of *E. coli*-associated enteric infections remains substantial, with recent analyses highlighting its continued contribution to diarrheal disease in low- and middle-income settings (2025).

Jamalludeen et al. demonstrated that prophylactic phage administration effectively mitigated *E. coli*-induced diarrhea in pigs, even at low fecal phage concentrations (< 103 PFU/g) (Jamalludeen et al., 2009). A three-phage cocktail exhibited pronounced efficacy, reinforcing phage therapy's utility in both preventive and therapeutic settings. Similarly, Vahedi et al. isolated a sewage-derived phage targeting enteropathogenic *E. coli* (EPEC) and confirmed its therapeutic potential in mice (Vahedi et al., 2018). Interestingly, phage monotherapy—whether administered preventively or curatively—outperformed both antibiotic treatment and combination therapies, resulting in complete pathogen clearance without compromising normal weight gain.

Clinical studies also have demonstrated the potential of phage therapy for *E. coli*-related diarrheal diseases. Bruttin et al. conducted a pioneering human trial involving 15 healthy male volunteers who received an oral dose of *E. coli* phage T4 (10^3^ PFU/mL) (Bruttin and Brüssow, 2005). While fecal phage was detectable in 50% of participants at 24 h post-administration, complete clearance occurred within 9 days with no reported adverse effects, confirming the safety profile. Notably, in the German outbreak of *E. coli* O104:H4 infections, environmental phage isolates showed effective lytic activity against the outbreak strain, contributing to successful epidemic control (Merabishvili et al., 2012).

### 2.3 Phage-based treatment of *L. monocytogenes*-induced diarrhea

*L. monocytogenes*, a facultative anaerobic pathogen, is a prominent foodborne bacterium prominent in various food products, including meat, vegetables, fruits, and dairy (Gana et al., 2024). The rise of antibiotic-resistant *L. monocytogenes* strains has significantly complicated clinical management, with treatment failures potentially leading to life-threatening outcomes (Skrobas et al., 2024).

In a murine listeriosis model, mice were orally challenged with 10^5^ CFU/mL of *L. monocytogenes*, followed by a 3-day course of six-phage cocktail therapy (10^5^ PFU/day) initiated 72 h post-infection (Mai et al., 2010). Phage therapy achieved a significant reduction in *L. monocytogenes* load compared to controls, with about an 80 CFU/g decrease in fecal bacterial counts. Treated mice maintained stable body weight, while control mice experienced approximately 10% weight loss due to diarrhea. Notably, phage-treated animals showed none of the adverse effects observed in antibiotic-treated controls, including increased watery stool and cecal distension. The detection of approximately 10^2^ CFU/g phage particles in cecal contents confirmed therapeutic phage amplification, demonstrating the efficacy of phage therapy in this diarrheal model.

### 2.4 Phage-based treatment of cholera

Cholera is an acute intestinal infectious disease caused by *Vibrio cholerae*, with its pathogenicity relying on the synergistic action of the toxin-co-regulated pilus (TCP) and cholera toxin (CT) (Yoon and Waters, 2019). This disease holds landmark significance in the early development of phage therapy. In the early 20th century, microbiologist Félix d'Herelle isolated specific phages by studying fecal samples from recovered cholera patients, providing crucial evidence for phage-mediated pathogen clearance (Sabino et al., 2020). Subsequent clinical trials demonstrated that oral phage therapy could dramatically reduce the mortality rate in early-stage cholera patients from 63 to 8%, showcasing its therapeutic potential (Table 2) (Summers, 1993).

**Table 2:** Case reports on the application of phage therapy against Cholera.

**Treatment agent**	**Model**	**Outcomes**	**References**
B1, B2, B3, B4 and B5	Rabbit	A 100-fold decrease in intestinal proliferation of *V. cholerae* after 24 h	Jaiswal et al., 2013
B1, B2, B3, B4 and B5	Mouse	Reducing the bacterial load of *V. cholerae* from ~10^7^ CFU/g	Jaiswal et al., 2014
ICP1, ICP2, ICP3	Mouse	Small-intestinal bacterial loads were reduced by ≥ 3 log_10_	Yen et al., 2017
ICP1, ICP2, ICP3, ICP4	Mouse	Exhibiting no weight loss or dehydration and maintaining normal bowel movements	Yen et al., 2017
ICP1, ICP2, ICP3, ICP4	Rabbit	Decreasing the amount of *V. cholerae* in the intestine by 10^7^ CFU/g	

In a recent study by Bhandare et al. researchers utilized a rabbit model of *V. cholerae* infection to evaluate the efficacy of oral phage therapy administered both prophylactically (pre-infection) and therapeutically (6 h post-infection) at a dose of 10^9^ PFU. Upon bacterial challenge, all experimental animals developed characteristic cholera-like symptoms, including significant cecal fluid accumulation and progressive diarrhea manifested through soft to watery stool consistency. Remarkably, phage-treated rabbits demonstrated complete restoration of normal fecal consistency following therapeutic intervention. Additionally, these treated animals maintained stable body temperatures and showed no observable behavioral abnormalities throughout the study period. Most notably, quantitative analysis revealed a substantial 100-fold reduction in intestinal *V. cholerae* colonization within 24 h of phage administration, accompanied by robust phage replication reaching titers of 10^7^ PFU/g in intestinal tissues (Bhandare et al., 2019).

Another complementary study, Jaiswal et al. demonstrated the therapeutic potential of a five-phage cocktail against *V. cholerae* infection in a rabbit model. When administered at 1 × 10^8^ PFU 6 h post-infection, the phage treatment produced remarkable protective effects. Treated animals maintained normal hydration status and stable body temperatures, in contrast to control rabbits that developed severe clinical manifestations. Quantitative analysis revealed a dramatic 85% reduction in cecal fluid accumulation (from 0.39 to 0.06 mL) and near-complete suppression of *V. cholerae* proliferation. Of note, prophylactic administration of the phage cocktail (10^9^ PFU) 6 h prior to bacterial challenge provided complete protection against cholera symptom development in juvenile rabbits (Jaiswal et al., 2013).

Furthermore, a five-phage cocktail demonstrated potent antibacterial efficacy in a *V. cholerae* mouse infection model. Phage therapy significantly suppressed bacterial proliferation, reducing intestinal *V. cholerae* loads from 7.1 × 10^6^ CFU/g to 9.1 × 10^3^ CFU/g on days 1 and 4 post-treatment. Concurrently, serum TNF-α levels decreased by 150 pg/mL after 4 days of therapy. Histopathological analysis revealed preserved intestinal architecture with minimal villous damage and no significant neutrophil infiltration in phage-treated mice, unlike the control group, which exhibited marked inflammatory and structural changes (Jaiswal et al., 2014). Prophylactic phage administration in both mouse and rabbit models substantially reduced intestinal *V. cholerae* colonization within 24 h post-infection (Yen et al., 2017). Control animals developed severe cholera-like symptoms, including cecal dilation, dehydration, and 10–12% weight loss, whereas phage-treated subjects maintained stable body weight, normal hydration, and regular bowel function, demonstrating the protective efficacy of phage therapy.

## 3 Targeted phage therapy against typhoid fever

Typhoid fever, caused by the invasive pathogen *Salmonella enterica* serovar Typhi, continues to pose a substantial global health challenge in endemic areas. Following intestinal colonization, *S. typhi* exhibits unique pathogenic capabilities to translocate across the intestinal epithelium, resulting in bloodstream invasion and systemic inflammatory responses. This predominantly waterborne/foodborne transmission cycle (Meiring et al., 2023) was first therapeutically targeted by Smith J. a century ago using intravenous phage administration (Smith, 1924), establishing an important proof-of-concept. Subsequent clinical advances by Knouf et al., demonstrated remarkable efficacy, reducing mortality rates from 14 to 5% in critically ill, comatose typhoid patients, highlighting phage therapy's potential against invasive salmonellosis (Table 3).

**Table 3:** Case studies on phage-based treatment of typhoid fever.

**Treatment agent**	**Model**	**Outcomes**	**References**
26 phage cocktails	Pig	Decreasing the colonization of *S. typhi* by 10^3^ CFU/g	Lee and Harris, 2001
Microencapsulated phages	Pig	Decreasing the colonization of *S. typhi* in the intestine by 10^8^ CFU/g	Wall et al., 2010
A 14-phage cocktail	Pig	Decreasing the colonization of *S. typhi* by 10^3^ CFU/g	Saez et al., 2011
*STWB21*	Mouse	The cure rates for the preventive and therapeutic groups were 66% and 33%	Callaway et al., 2011
Microencapsulated phages	Pig	Reducing *S. typhi* colonization by 95% and 90%	Callaway et al., 2011

Mondal et al., recently identified STWB21, an environmentally stable lytic phage isolated from lake water, which demonstrated treatment-timing-dependent efficacy in a *S. typhi*-infected murine model. Prophylactic administration demonstrated superior efficacy with a 66% cure rate compared to therapeutic intervention's 33% success rate, likely attributable to STWB21's capacity to prevent initial *S. typhi* intestinal colonization (Mondal et al., 2022). Research indicated that prophylactic STWB21 administration was more effective than therapeutic application, likely attributable to its ability to suppress *S. typhi* intestinal colonization. Histopathological analysis demonstrated significantly lower *S. typhi* burdens in the liver and spleen of STWB21-treated mice compared to controls. Electron microscopy further revealed severe hepatic abscess formation and venous inflammation in control animals, whereas STWB21-treated mice maintained normal tissue architecture with only a moderate increase in mitochondrial and lysosomal activity (Mondal et al., 2023).

Three independent studies using *S. typhi*-infected porcine models demonstrated rapid and significant reductions in bacterial loads following phage therapy (Callaway et al., 2011; Lee and Harris, 2001; Saez et al., 2011). Saez et al., reported complete bacterial clearance within 6 h of oral administration of microencapsulated phages, with pathogenic bacteria detectable only in control animals upon analysis of cecal and colonic contents (Saez et al., 2011). Wall et al. observed 95 and 90% reductions in *S. typhi* colonization in the cecum and ileum, respectively, using similar microencapsulated phage formulations (Wall et al., 2010). In a parallel study by Callaway et al., pigs treated with a phage cocktail (3 × 10^9^ PFU) at 24- and 48-h post-infection exhibited fecal *S. typhi* levels tenfold lower than controls by 48 h (Callaway et al., 2011).

## 4 Phage-based therapeutics for intestinal inflammation

Clinically significant enteropathogens; including *E. coli, C. difficile*, and *Y. enterocolitica*, employ distinct virulence mechanisms to establish persistent intestinal inflammation (Shuwen and Kefeng, 2022). In this context, phage therapy emerges as a transformative therapeutic strategy, offering targeted elimination of pathogenic bacteria while maintaining commensal microbiota homeostasis, thereby overcoming the limitations of conventional antibiotics that often aggravate microbial dysbiosis and providing a precision approach to interrupt this self-perpetuating pathogenic cycle (Arthur et al., 2012).

*Y. enterocolitica*, a zoonotic enteropathogen, induces intestinal inflammation upon host colonization (Leon-Velarde et al., 2019). Xue et al., developed an intestinal-targeting lytic phage and evaluated its efficacy in a murine model (Xue et al., 2020). Following oral challenge with 2 × 108 CFU *Y. enterocolitica*, administration of a single phage dose (10^9^ PFU/mL) at 6 h post-infection achieved complete bacterial clearance in 33% of mice, a 4-log reduction in colonic/cecal bacterial loads (from 107 to 10^3^ CFU/g), and sustained suppression for 144 h. Phage-treated mice also showed significantly lower pro-inflammatory cytokine levels, demonstrating therapeutic potential against Yersinia-induced enteritis.

*C. difficile* has been widely recognized as a major pathogen driving intestinal inflammation (Dong et al., 2023), exerting its pathogenic effects through gut microbiota disruption and modulation of host immune responses. In a recent study, Shan et al. demonstrated the therapeutic potential of phage therapy using an *in vitro* colon cell model infected with *C. difficile*, where single-phage treatment successfully eliminated adherent bacteria without causing collateral damage to host cells (Shan et al., 2018), highlighting its safety profile for potential clinical applications. *In vivo* studies have yielded promising results. Ramesh et al. administered phage therapy (108 PFU/mL) by oral gavage in a hamster model of colitis. While all control animals succumbed within 72 h with severe cecal pathology (bleeding and swelling), the phage-treated group showed significantly improved survival, with 2 × 10^4^ PFU of phages recovered from the cecum (Ramesh et al., 1999). Selle et al. employed an innovative approach by engineering a phage using the Type I CRISPR-Cas system to target *C. difficile*-induced intestinal inflammation. In their mouse model, a single dose of 10^9^ PFU of the engineered phage administered on day 4 post-infection (initiated with 105 CFU of *C. difficile*) reduced tissue damage scores by 4 points compared to controls. Notably, high phage titers (108 PFU/g) persisted in feces 4 days post-treatment, accompanied by significant improvements in cecal inflammation and bacterial clearance (Selle et al., 2020).

## 5 Bacteriophage strategies to combat sepsis-associated infections

Sepsis, a life-threatening syndrome characterized by organ dysfunction, represents a critical global health challenge with persistently high mortality rates (Rudd et al., 2020). Of particular clinical relevance is gut-origin sepsis (Assimakopoulos et al., 2018), a distinct subtype that arises when intestinal pathogens compromise the mucosal barrier integrity, leading to both structural damage and functional impairment of the intestinal epithelium (Amornphimoltham et al., 2019). This reach initiates a pathogenic cascade involving bacterial translocation, which subsequently evokes a robust systemic inflammatory response that may culminate in intestinal failure and progressive multiple organ dysfunction syndrome (MODS) (Haussner et al., 2019). Mounting experimental and clinical evidence now underscores the pivotal contribution of gut microbiota dysbiosis to the pathogenesis of gut-derived sepsis, thereby establishing a compelling therapeutic rationale for investigating phage-based interventions as a precision antimicrobial strategy (Magnan et al., 2023).

Intestinal colonization by *Pseudomonas aeruginosa* can rapidly progress to life-threatening sepsis with high mortality rates (Tabarani and Baker, 2022). It is demonstrated that a single oral dose of lysogenic phage (10^10^ PFU/mL) significantly improved survival outcomes. Phage therapy increased survival rates by 66.7% compared to saline-treated controls while simultaneously reducing *P. aeruginosa* burden in the liver and spleen by 1 log CFU/g. Notably, phage-treated animals showed a 4–5-fold reduction in proinflammatory cytokine levels (Watanabe et al., 2007). In a complementary study, Prokopczuk et al., developed an engineered Pf phage that achieved >4-log CFU/g reduction in bacterial load in murine infection models. Most strikingly, while untreated controls exhibited near-complete (100%) mortality, all Pf-treated animals survived the entire observation period. Histopathological evaluation further confirmed the phage's ability to prevent bacterial dissemination to secondary organs (liver and spleen) (Prokopczuk et al., 2023).

*Enterococcus faecium* has evolved from a commensal organism to a leading nosocomial pathogen, with surveillance data demonstrating a striking increase in its association with life-threatening infections since the 1980s (García-Solache and Rice, 2019). This epidemiological shift is particularly concerning in cases of *E. faecium*-induced sepsis, where therapeutic options are severely constrained by both intrinsic high mortality rates and the expanding global prevalence of vancomycin-resistant (VREfm) and multidrug-resistant strains (Cattaneo et al., 2021; Torres et al., 2018). Stellfox et al., report the successful use of integrative phage therapy to treat recurrent *E. faecium* bacteremia from persistent gut colonization in an immunocompromised patient. The therapeutic protocol combined conventional antibiotics (vancomycin-daptomycin) with adjunctive phage therapy administered at 1 × 10^9^ PFU via optimized dual-route delivery (oral and intravenous) (Stellfox et al., 2024). This therapeutic strategy achieved two essential clinical outcomes; complete sepsis resolution with blood culture sterilization within 27 days alongside durable prevention of recurrent bacteremia through sustained phage maintenance therapy.

## 6 Bridging the gap in gut phage therapy

Antibiotic misuse has fueled the rise of multidrug-resistant bacteria, posing a grave threat to public health worldwide. Equally concerning is the collateral damage from broad-spectrum antibiotics, which devastate the gut microbiota. This destruction triggers a chain of events: the ecological balance is disrupted, the gut barrier is compromised, and susceptibility to opportunistic infections rises (Singha et al., 2023). Unlike antibiotic treatment, which frequently causes gut microbiota dysbiosis in infected animals, phage therapy confers the added benefit of maintaining a relatively normal microbial community structure, a protective effect demonstrated in a *C. difficile* colitis model by improved survival, reduced intestinal damage, and minimal impact on the resident gut microbiota (Gundersen et al., 2023; Raeisi et al., 2023). Gut microbiota dysbiosis is a key driver of intestinal inflammation, and by precisely eliminating inflammatory pathogens such as *Yersinia, Klebsiella pneumoniae*, and *C. difficile*, bacteriophages reduce local and systemic pro-inflammatory cytokine (e.g., TNF-α) levels, thereby alleviating inflammation and promoting tissue repair (Wang et al., 2022; Federici et al., 2022). This causal strategy ultimately breaks the vicious cycle of pathogen-driven inflammation at its root.

Phage therapy has demonstrated potential for treating intestinal infections in animal models, yet its clinical translation faces four major challenges. First, as living biological entities, phages exhibit complex pharmacokinetics with significant inter-individual variability. Oral administration is susceptible to gastrointestinal environmental factors, and efficacy heavily depends on their replication capability at the infection site, making standardized dosing difficult (Benyamini, 2024). Second, the host immune system can generate neutralizing antibodies, particularly during systemic or repeated administration, which substantially compromises subsequent treatment efficacy (Van Nieuwenhuyse et al., 2022). Third, the production of “phage cocktails” faces challenges in achieving batch-to-batch consistency, and the absence of globally unified quality control standards hinders regulatory approval and large-scale application. Fourth, existing animal models have inherent limitations and cannot fully replicate the complex environment of human intestinal infections, thereby limiting the predictive value of preclinical data (Browne et al., 2016).

Addressing the clinical translation challenges of phage therapy necessitates an integrated multi-faceted approach. Key priorities include developing intelligent delivery systems, such as pH-responsive microcapsules, to safeguard phages during gastrointestinal transit and enable site-specific release, thereby augmenting their colonization and replication efficacy at infection sites (Vinner et al., 2019). Concurrently, phage engineering through gene editing and surface modifications like PEGylation can mitigate neutralization by host antibodies and extend systemic circulation (Gordillo Altamirano and Barr, 2019). Manufacturing standardization requires implementing Quality by Design principles and synthetic biology techniques to ensure consistent production of phage cocktails with reproducible therapeutic outcomes (Malik, 2021). Furthermore, establishing human-relevant models—including humanized intestinal organoids integrated with multi-omics platforms—will strengthen the predictive validity of translational studies. Collectively, these strategies will accelerate the transition of phage therapy from experimental research to clinical implementation (Shield et al., 2021).

## 7 Conclusion

This review systematically examines phage therapy against intestinal infections in animal models, including diseases such as diarrhea, enteritis, and systemic sepsis caused by pathogens like *Shigella*, pathogenic *E. coli, L. monocytogenes, V. cholerae, S. typhi* and *C. difficile*. Given the global rise of antibiotic resistance, developing such alternative therapies is critically important.

The results consistently demonstrate that phage therapy effectively clears targeted pathogens, alleviates intestinal inflammation, and preserves barrier integrity. It outperformed traditional antibiotics across multiple models without inducing the microbiota dysbiosis frequently associated with them. A key insight is that prophylactic administration generally affords stronger protection than therapeutic intervention. Furthermore, combining rationally engineered phages with polyvalent cocktail formulations produces synergistic effects, significantly boosting the therapeutic potential.

While persistent challenges—such as narrow host range, evolving bacterial resistance, and the complex gut environment—remain, advances in optimized delivery systems, precision phage engineering, and multi-omics integration are progressively addressing these limitations. These developments establish phage therapy as a key strategy against multidrug-resistant intestinal infections and provide a solid foundation for clinical translation. Future efforts should focus on developing novel engineering approaches to broaden host range and counter resistance, elucidating the multifaceted interactions between phages, host immunity, and gut microbiota, and optimizing clinical dosing regimens and formulations to facilitate well-controlled human trials.
